# ECDC’s latest publications

**DOI:** 10.2807/1560-7917.ES.2017.22.39.170928-3

**Published:** 2017-09-28

**Authors:** Eurosurveillance editorial team

**Affiliations:** 1 European Centre for Disease Prevention and Control (ECDC), Stockholm, Sweden

**Keywords:** infectious diseases, public health, Europe

**Rapid risk and outbreak assessments**

Increase of Legionnaires' disease in EU travellers returning from Dubai since October 2016

Date of publication: 21 September 2017

https://ecdc.europa.eu/en/publications-data/rapid-risk-assessment-increase-legionnaires-disease-eu-travellers-returning-dubai

Multiple reports of locally-acquired malaria infections in the EU

Date of publication: 20 September 2017

https://ecdc.europa.eu/en/publications-data/rapid-risk-assessment-multiple-reports-locally-acquired-malaria-infections-eu

Clusters of autochthonous chikungunya cases in Italy

Date of publication: 14 September 2017

https://ecdc.europa.eu/en/publications-data/rapid-risk-assessment-clusters-autochthonous-chikungunya-cases-italy

Hurricane Irma: risk of communicable diseases in the affected countries 

Date of publication: 11 September 2017

https://ecdc.europa.eu/en/publications-data/rapid-risk-assessment-hurricane-irma-risk-communicable-diseases-affected

Cluster of autochthonous chikungunya cases in France

Date of publication: 24 August 2017

https://ecdc.europa.eu/en/publications-data/rapid-risk-assessment-cluster-autochthonous-chikungunya-cases-france

Public health risks related to communicable diseases during the Hajj 2017, Saudi Arabia, 30 August - 4 September 2017 

Date of publication: 10 August 2017

https://ecdc.europa.eu/en/publications-data/rapid-risk-assessment-public-health-risks-related-communicable-diseases-during

Cyclospora infections in European travellers returning from Mexico 

Date of publication: 21 July 2017

https://ecdc.europa.eu/en/publications-data/rapid-risk-assessment-cyclospora-infections-european-travellers-returning-mexico

Multi-country outbreak of Salmonella Enteritidis phage types 56 and 62, MLVA profile 2-11-3-3-2 and 2-12-3-3-2 infections 

Date of publication: 20 July 2017

https://ecdc.europa.eu/en/publications-data/rapid-risk-assessment-multi-country-outbreak-salmonella-enteritidis-phage-types

Influenza A(H7N9) virus in China - implications for public health 

Date of publication: 3 July 2017

https://ecdc.europa.eu/en/publications-data/influenza-ah7n9-virus-china-implications-public-health-7th-update-3-july-2017

Hepatitis A outbreak in the EU/EEA mostly affecting men who have sex with men

Date of publication: 28 June 2017

https://ecdc.europa.eu/en/publications-data/rapid-risk-assessment-hepatitis-outbreak-eueea-mostly-affecting-men-who-have-sex ****

**Technical reports**

Assessment of infection control, hospital hygiene capacity and training needs in the European Union 

Date of publication: 6 September 2017

https://ecdc.europa.eu/en/publications-data/assessment-infection-control-hospital-hygiene-capacity-and-training-needs

Utilising social media to support HIV/STI prevention: evidence to inform a handbook for public health programme managers 

Date of publication: 27 July 2017

https://ecdc.europa.eu/en/publications-data/utilising-social-media-support-hivsti-prevention-evidence-inform-handbook-public

Seasonal influenza vaccination in Europe: vaccination recommendations and coverage rates in the EU Member States for eight influenza seasons 

Date of publication: 20 July 2017

https://ecdc.europa.eu/en/publications-data/seasonal-influenza-vaccination-europe

Gap analysis on securing diphtheria diagnostic capacity and diphtheria antitoxin availability in the EU/EEA 

Date of publication: 12 July 2017

https://ecdc.europa.eu/en/publications-data/gap-analysis-securing-diphtheria-diagnostic-capacity-and-diphtheria-antitoxin

**Scientific advice**

Expert opinion on rotavirus vaccination in infancy 

Date of publication: 7 September 2017

https://ecdc.europa.eu/en/publications-data/expert-opinion-rotavirus-vaccination-infancy

Zika virus and safety of substances of human origin: a guide for preparedness activities in Europe — first update

Date of publication: 16 August 2017

https://ecdc.europa.eu/en/publications-data/zika-virus-and-safety-substances-human-origin-guide-preparedness-activities-0

Expert opinion on neuraminidase inhibitors for the prevention and treatment of influenza - review of recent systematic reviews and meta-analyses 

Date of publication: 14 August 2017

https://ecdc.europa.eu/en/publications-data/expert-opinion-neuraminidase-inhibitors-prevention-and-treatment-influenza-review

**Technical documents**

ECDC tool for the prioritisation of infectious disease threats 

Date of publication: 7 August 2017

https://ecdc.europa.eu/en/publications-data/ecdc-tool-prioritisation-infectious-disease-threats

Utilising social media for HIV/STI prevention programmes among young people 

Date of publication: 24 July 2017

https://ecdc.europa.eu/en/publications-data/utilising-social-media-hivsti-prevention-programmes-among-young-people

**Surveillance reports**

Gonococcal antimicrobial susceptibility surveillance in Europe, 2015 

Date of publication: 24 August 2017

https://ecdc.europa.eu/en/publications-data/gonococcal-antimicrobial-susceptibility-surveillance-europe-2015

Influenza virus characterisation, Summary Europe, June 2017

Date of publication: 22 August 2017

https://ecdc.europa.eu/en/publications-data/influenza-virus-characterisation-summary-europe-june-2017

ECDC/EFSA/EMA second joint report on the integrated analysis of the consumption of antimicrobial agents and occurrence of antimicrobial resistance in bacteria from humans and food-producing animals 

Date of publication: 27 July 2017

https://ecdc.europa.eu/en/publications-data/ecdcefsaema-second-joint-report-integrated-analysis-consumption-antimicrobial

Hepatitis E in the EU/EEA, 2005-2015

Date of publication: 11 July 2017

https://ecdc.europa.eu/en/publications-data/hepatitis-e-eueea-2005-2015

ECDC communicable disease threats report

Date of publication: every Friday

https://ecdc.europa.eu/en/threats-and-outbreaks/reports-and-data/weekly-threats

**Corporate publication**

Annual report of the Director - 2016 

Date of publication: 21 July 2017

https://ecdc.europa.eu/en/publications-data/annual-report-director-2016

